# Tetrandrine Ameliorates Airway Remodeling of Chronic Asthma by Interfering TGF-*β*1/Nrf-2/HO-1 Signaling Pathway-Mediated Oxidative Stress

**DOI:** 10.1155/2019/7930396

**Published:** 2019-11-03

**Authors:** Yiping Lin, Jingchan Yao, Meiling Wu, Xiaoqian Ying, Mingxing Ding, Yanli Wei, Xiaoyan Fu, Wei Feng, Yunguang Wang

**Affiliations:** ^1^Department of Pediatrics, School of Medicine, Jinhua Polytechnic, Jinhua 321007, Zhejiang, China; ^2^Department of Pharmacology, School of Medicine, Jinhua Polytechnic, Jinhua 321007, Zhejiang, China; ^3^Department of Histologic, School of Medicine, Jinhua Polytechnic, Jinhua 321007, Zhejiang, China; ^4^Department of Radiation Oncology, Institute of Cancer Research and Basic Medical Sciences of Chinese Academy of Sciences, Cancer Hospital, University of Chinese Academy of Sciences, Zhejiang Cancer Hospital, Hangzhou 310022, Zhejiang, China; ^5^Institute of Nuclear-Agricultural Sciences, Zhejiang University, Hangzhou 310058, Zhejiang, China

## Abstract

**Background:**

Imbalanced oxidative stress and antioxidant defense are involved in airway remodeling in asthma. It has been demonstrated that Tetrandrine has a potent role in antioxidant defense in rheumatoid arthritis and hypertension. However, the correlation between Tetrandrine and oxidative stress in asthma is utterly blurry. This study aimed to investigate the role of Tetrandrine on oxidative stress-mediated airway remolding.

**Materials and Methods:**

Chronic asthma was established by ovalbumin (OVA) administration in male Wistar rats. Histopathology was determined by HE staining. Immunofluorescence was employed to detect the expression of *α*-SMA and Nrf-2. Level of oxidative stress and matrix metalloproteinases were examined by ELISA kits. Cell viability and cell cycle of primary airway smooth muscle cells (ASMCs) were evaluated by CCK8 and flow cytometry, respectively. Signal molecules were detected using western blot.

**Results:**

Tetrandrine effectively impairs OVA-induced airway inflammatory and airway remodeling by inhibiting the expression of CysLT1 and CysLTR1. The increase of oxidative stress and subsequent enhancement of MMP9 and TGF-*β*1 expression were rescued by the administration of Tetrandrine in the rat model of asthma. In in vitro experiments, Tetrandrine markedly suppressed TGF-*β*1-evoked cell viability and cell cycle promotion of ASMCs in a dose-dependent manner. Furthermore, Tetrandrine promoted Nrf-2 nuclear transcription and activated its downstream HO-1 in vivo and in vitro.

**Conclusion:**

Tetrandrine attenuates airway inflammatory and airway remodeling in rat model of asthma and TGF-*β*1-induced cell proliferation of ASMCs by regulating oxidative stress in primary ASMCs, suggesting that Tetrandrine possibly is an effective candidate therapy for asthma.

## 1. Introduction

Asthma, a heterogeneous respiratory disorder, is disturbuted in about 300 million people around the world [1]. In China, more than 30 million people suffer from asthma, and asthma-rated mortality is the highest in the world [2]. Airway remodeling has been regarded as the main reason of airway hyperresponsiveness (AHR) and lung function disorder of asthma, which is closely associated with subepithelial fibrosis, hyperplasia of airway smooth muscle cells (ASMCs), and excessive extracellular matrix (ECM) [3, 4]. Unfortunately, current clinical drugs have little effects on the improvement of airway remodeling.

Amount of evidence shows that transforming growth factor-*β* (TGF-*β*) plays a pivotal role in airway remodeling of asthma [5–7]. TGF-*β*1 prominently stimulates airway inflammation by upregulating the expression of IL-8, COX-2, and PGE2 [8]. TGF-*β* also induces airway wall thickening by increasing the proliferation and differentiation ability of ASMCs via affecting MAP kinases activity and Ca^2+^ homeostasis [9, 10]. TGF-*β*1 can regulate the expression of IL-4, IL-5, IL-13, and eotaxin, resulting in airway inflammation and pulmonary fibrosis of chronic asthmatic mouse [11]. Isoproterenol- (ISO-) induced relaxation of ASMCs is impaired by TGF-*β*1/Smad2/3 signal transduction via modulating intracellular cAMP levels [12]. The TGF-*β*/smad2 pathway also aggravates nonspecific hyperreactivity by regulating constriction of AMSCs [13]. These studies imply that TGF-*β* might be able to interfere in the progress of asthma by affecting biological function of ASMCs.

Besides, oxidative stress dysfunction is another key trigger of airway remodeling. A previous study has demonstrated that reactive oxygen species- (ROS-) evoked oxidative stress stimulates matrix metalloproteinases (MMPs) expression resulting in the remodel of airway smooth muscle [14]. As a potent antioxidant factor, nuclear factor erythroid 2-related factor 2 (Nrf-2) level is closely correlated with the progression of asthma, and antioxidant markers including superoxide-dismutase (SOD) and glutathione peroxidase (GPX) exhibit low expression in severe bronchial asthma which might be associated with Nrf-2 [15]. Vitamin E isoform *γ*-tocotrienol, with superior antioxidant properties than vitamin E, abates oxidative damage and hyperresponsiveness (AHR) by augmenting Nrf-2 nuclear level in allergic asthma [16]. It is confirmed that oxidative stress enlarges the effects of TGF-*β*1 signaling-induced cell viability of AMSCs [17]. Importantly, antioxidant regulation has become a hot topic in asthma research in recent years [18]. Therefore, targeting oxidative stress possibly is the potential therapeutic strategy of airway remodeling-mediated asthma.

Tetrandrine (Tet) extracted from the root of *Stephania tetrandra* S. Moore is a common bisbenzylisoquinoline alkaloid [19]. Mounting studies have confirmed that Tetrandrine alleviates the articular inflammatory response by inhibiting the expression of IL-6, IL-1*β*, and TNF-*α* in macrophage and chondrocyte [20]. Tetrandrine increases the expression of antioxidative enzymes such as SOD and GSH, which relieve monocrotaline-induced pulmonary arterial hypertension [21]. Among respiratory diseases, Tetrandrine reduces the secretion of inflammatory factors including IL-2, IL-4, and IFN-*γ* in asthmatic patients leading to the improvement of symptoms [22]. Isotetrandrine, an isomeride of Tetrandrine, is able to ameliorate tert-butyl hydroperoxide-induced oxidative damage of liver cancer cells through dissociating Nrf2-Keap1 complex [23]. However, the role of Tetrandrine on oxidative stress-mediated airway remodeling and subsequent development of asthma is still unclear.

Herein, we investigated that Tetrandrine administration notably inhibited pulmonary inflammatory and airway remodeling in vivo. Treatment with Tetrandrine also induced ASMC cells cycle arrest and inhibited cell growth of ASMCs by interfering in the TGF-*β*1/Nrf-2/HO-1 signaling pathway. These results suggest a potential therapeutic action of Tetrandrine in airway remodeling-mediated asthma.

## 2. Materials and Methods

### 2.1. Asthma Model Establishment and Animal Treatment

#### 2.1.1. Animals

6-week-old male Wistar rats were randomly divided into 3 groups including control, asthma, and asthmatic with tetrandrine treatment groups. All animal care procedures were approved by Laboratory Animals of Jinhua Polytechnic.

#### 2.1.2. Animal Model and Study Design

For the isolation of ASMCs, acute asthmatic rat models were established by ovalbumin (OVA) inhalation. Briefly, rats were intraperitoneally injected with 1 mg OVA (grade V; Sigma-Aldrich; St. Louis, MO) containing 5% Al (OH)_3_ (Thermo Scientific) in 1 ml saline solution on days 1, 8, and 15. On days 16–20, rats were challenged with 1 mg OVA adsorbed in 1 ml sterile saline solution through an ultrasonic nebulizer for 30 min each time. Control rats were injected and inhalated with equivalent and isopyknic saline. From day 16, treatment groups were given 100 mg/kg Tetrandrine by gavage 2 h before OVA atomization. Control and asthmatic model groups received equivalent and isopyknic saline.

For a chronic asthma model, rats were divided into three groups including control plus saline, asthma plus saline, and asthma plus Tetrandrine groups. In brief, rats were also challenged with saline and 1 mg/ml OVA solution through an ultrasonic nebulizer for 30 min each time, 3 times per week for 8 weeks. Rats challenged with OVA were divided randomly into two groups. 2 h before nebulization, the control group was given saline by gavage. And the two group asthmatic rats were administrated with equivalent and isopyknic saline and 100 mg/kg Tetrandrine by gavage once a day for 8 weeks, respectively. After completing model creation, lung tissue was obtained from each group and stored at −80°C. Fresh lung tissue was used to isolate primary airway smooth muscle cells and detect the levels of biochemical indicator. The other tissue was fixed in 4% paraformaldehyde overnight at 4°C for HE and immunostaining.

#### 2.1.3. Histological Staining

For observing the morphology of lung tissue in different groups, lung tissue were fixed in 4% paraformaldehyde and embedded in paraffin. Paraffin-embedded specimens were cut into 5 *μ*m sections and dewaxed in xylene and rehydrated in a 100% to 50% ethanol gradient. Then, the sections were stained with hematoxylin (CTS-1099, MXB Biotechnologies, China) for 1 minute and washed with running water for 2 minutes. After being differentiated in hydrochloric acid-ethanol for 15 secs, samples were washed with ammonia water for 10 secs and stained with eosin (G1100, Solarbio, China) for 5 minutes. Then, the sections were mounted with glycerinum and the morphology was observed under a biological inverted microscope (IX51, Olympus, Japan).

#### 2.1.4. Isolation and Authentication of ASMCs

The rat trachea was separated from lung tissue followed by inner membrane removal. Then, airway smooth muscle was cut from the trachea and washed with DMEM (11320-033, GIBCO, USA) containing penicillin-streptomycin (15070-063, GIBCO, USA) for three times. Smooth muscle was digested with 0.1% of type IV collagenase in 37°C for 30 min. After reaction was aborted by DMEM containing 10% FBS (10091-148, GIBCO, USA), the supernatant containing cell debris were filtered with 200 mesh and cells were cultured in 5% CO_2_ incubator at 37°C. Passage 4 of ASMCs was used for the next experiment before a-SMA immunofluorescence identification.

#### 2.1.5. CCK8 Assay

Primary ASMCs were seeded in a 96-well plate at the density of 5 × 10^3^ cells/well. After culturing for 24 h, cells were treated with TGF-*β*1 (10 *μ*g/L) or TGF-*β*1 plus Tetrandrine (10, 20, or 40 *μ*g/ml) for 48 h. Then, cells viability was detected by adding 10 *μ*L CCK8 (MCE, HY-K0301, US) and incubated for additional 4 h. The absorbance was measured at the wavelength of 450 nm by a Multiscan plate reader (MK3; Thermo Fisher Scientific, Waltham, MA, USA).

#### 2.1.6. Cell Cycle Detection

ASMCs were plated in 6-well plate at the density of 2 × 10^5^ cells/well and cultured overnight in 5% CO_2_ incubator at 37°C. After treatment with TGF-*β*1 (10 *μ*g/L) or Tetrandrine for 24 h, each group cells were harvested and fixed in 75% ethanol at 4°C overnight. Then cells were washed with precooled PBS for 3 times, followed by PI (50 *μ*g/ml) and RNase A (50 *μ*g/ml) incubation for 30 min at 37°C in dark. Eventually, the cell cycle was analyzed by flow cytometry (BECKMAN, CytoFLEX).

#### 2.1.7. Western Blotting

Western blot assays were proceeded as previously described [24]. Briefly, 40 *μ*g total protein was separated by 10% SDS-PAGE and transferred to an activated PVDF membrane (IPVH00010, Millipore, Thermo Scientific, USA). The membrane was immersed in 5% fat-free milk at room temperature for 1 h and incubated with primary antibodies against CysLT1 (ab151484, Abcam, USA), CysLTR1 (a5393, Boster, China), Nrf-2 (ab31163, Abcam, USA), and HO-1 (ab13243, Abcam, USA) at 4°C overnight. *β*-actin (BM0627, Boster, China) served as the internal control. After incubation with Goat anti-Rabbit IgG Secondary Antibody, HRP (BA1054, BOSTER, China), or Goat anti-Mouse IgG Secondary Antibody, HRP (BA1051, BOSTER, China) at room temperature for 1 h, the membrane was washed with TBST for 3 times. The proteins were visualized after incubating with ECL (NCI5079, Thermo Scientific, USA) using the Imagequant LAS 4000 mini machine (GE Healthcare Life Sciences, USA). All samples were performed at least 3 independent experiments.

#### 2.1.8. RNA Extraction and qRT-PCR

Total RNA was extracted with Trizol Reagent (15596026, Invitrogen, USA) according to the manufacture's instruction. The synthesis of first cDNAs was performed by PCR reaction using the PrimeScriptTM RT reagent kit (Fermentas, Thermo Scientific K1622). Then, qPCR was proceeded using SYBR Premix Ex Taq II by the following procedures: 95°C for 30 s and 40 total cycles of 95°C for 15 s, 60°C for 1 min, and 60°C for 45 s. Three replicates were set for each sample. Relative mRNA levels of all genes were normalized to control GAPDH. The relative expression of target genes was calculated by using the comparative 2^−ΔΔCt^ method. The primer sequences are shown in Table 1.

#### 2.1.9. Immunofluorescence

Slides were then incubated with specific primary antibodies against a-SMA (rat, Ab5694, 1 : 200, Abcam, USA) and Nrf2 (Ab62352, 1 : 200, Abcam, USA) at 4°C overnight. Next day, after washing with 1x PBS for 5 min/3times, slides were incubated with secondary antibody (1 : 500, Boster, China) for 1 h at room temperature. Then, samples were stained with DAPI (AR1177, BOSTER, China) and sealed in antifade fluorescence mounting medium (AR1109, BOSTER, China) with coverslips. The detection of a-SMA and Nrf2 proteins was performed under a fluorescence microscope (IX71, Olympus, Japan). Nrf-2 positive cells were counted using Image pro plus software. The percentage of Nrf-2 in the nucleus was quantitatively analyzed by the ratio of nuclear Nrf-2 positive cells to total cells in each field.

#### 2.1.10. Oxidative Stress Detection and ELISA Assay

Levels of oxidative stress and cysteinyl leukotrienes were measured by ELISA assay. In brief, lung tissue was ground in 1 ml physiological saline solution. After centrifugation at 2500 rpm for 10 min, the supernatants of the homogenate were used to detect the content of glutathione disulfide (GSSG), glutathione (GSH), glutamyl cysteine synthase (*γ*-GCS), cysteinyl leukotrienes 1 (CysLT1), and cysteinyl leukotriene receptor 1 (CysLTR1). GSSG/GSH (Nanjing Jiancheng Bioengineering Institute, A061-1, China) and *γ*-GCS were measured by total glutathione/oxidized glutathione assay kits (Nanjing Jiancheng Bioengineering Institute, A091-1, China) and gamma-glutamylcysteine synthetase kit (Nanjing Jiancheng Bioengineering Institute, A091-1, China). CysLT1/CysLTR1 (CUSABIO, CSB-EL006465RA, US) was detected by ELISA kits according to the manufacture's instruction.

#### 2.1.11. Statistical Analyses

All experiments were repeated at least 3 times. HE and IF staining results were analyzed using Image J software. Numerical data are presented as mean ± SD, and these data were statistically analyzed by a one-tailed Student's *t* test or one-way ANOVA by using GraphPad Prism 5.0 software. Statistically significant differences were accepted at *P* < 0.05.

## 3. Results

### 3.1. Tetrandrine Reverses Ovalbumin- (OVA-) Induced Inflammation and Airway Remodeling in Rat Model with Asthma

**Figure 1:**
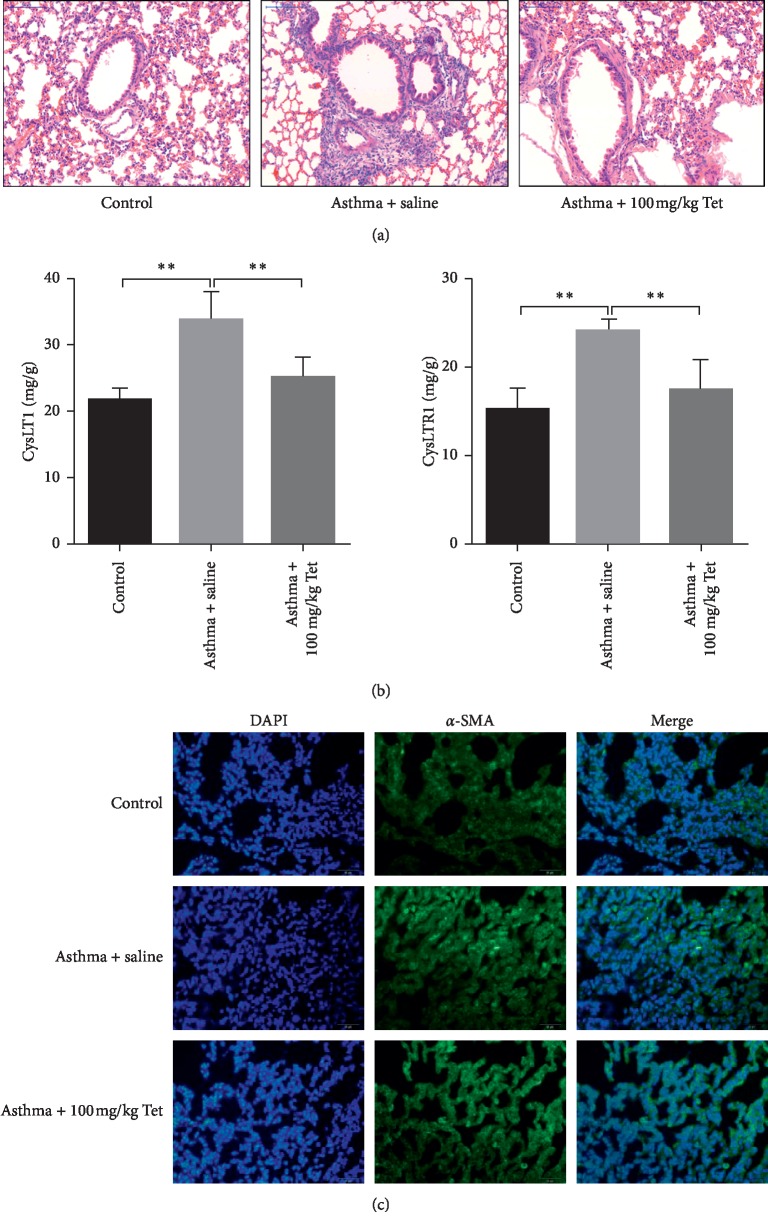
Effects of Tetrandrine on pulmonary pathology and airway remodeling in mouse with asthma. (a) H&E staining of lung tissues from control, control + tet, asthma, and asthma + tet; Tet: Tetrandrine. (b) The expression of CysLT1 and CysLTR1 analyzed by ELISA assay. *p* < 0.0001, *F* value: 65.4 and 56.11. (c) Images of *α*-SMA protein expression in different groups determined by immunofluorescence assay. Multiple images were taken, and representative images were presented. ^*∗∗*^Asthma group vs. control, control + tet vs. asthma + tet and asthma + tet group vs. model group. Scale bar: 50 *μ*m. Data represent the mean ± SD of three experiments, each performed in triplicate. ^*∗∗*^*p* < 0.01.

To evaluate the role of Tetrandrine on the progression of asthma, OVA-sensitized rat models with asthma were treated with Tetrandrine (100 mg/kg) for successive 8 weeks. Through HE staining, we observed OVA evoked the airway wall thickening and inflammatory aggressive around the trachea compared to control rats (Figure 1(a), the left two images). However, Tetrandrine exposure obviously rescued OVA-mediated alveolar inflammatory infiltration and basement membrane thickness (Figure 1(a), the third image). CysLT1, as a potent inflammatory lipid mediator, stimulates inflammation response in airway through binding to its receptor CysLTR1 [25]. In our data, we discovered that both the expressions of CysLT1 and CysLTR1 were significantly increased under the stimulation of OVA compared with control, which were obviously reduced in the presence of Tetrandrine administration (Figure 1(b)). Besides, IF staining using *α*-SMA (airway remodeling marker) further confirmed that the expression and description of *α*-SMA in airway smooth muscle were robustly enhanced in asthma model group and Tetrandrine-treated group, while OVA-mediated increase of *α*-SMA was observably decreased in the condition of Tetrandrine, implying that Tetrandrine might reverse OVA-induced airway remodeling (Figure 1(c)). However, Tetrandrine treatment only had no impact on lung histology in the control group. These observations indicate that Tetrandrine effectively relieves OVA-induced airway inflammatory and airway remodeling.

### 3.2. Tetrandrine Impairs TGF-*β*1-Induced Cell Viability of ASMCs

**Figure 2:**
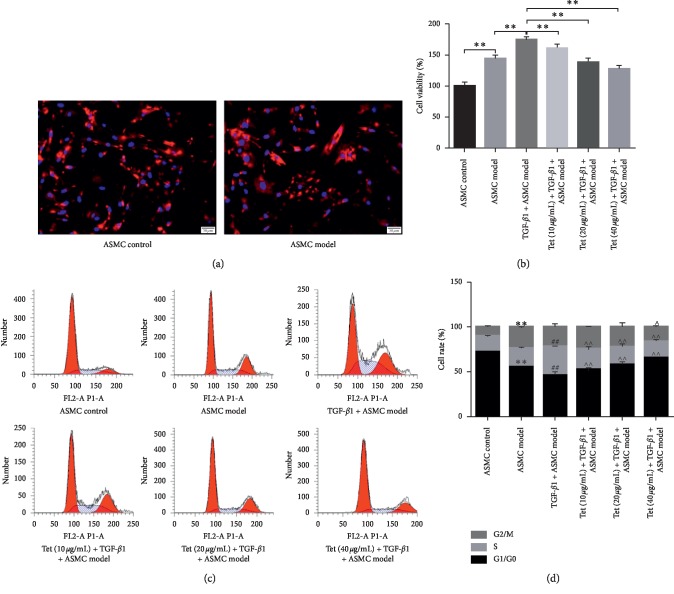
Effects of Tetrandrine on primary ASMCs proliferation. (a) Verification of primary ASMCs measured by IF assay. Red: *α*-actin; blue: DAPI. Scale bar: 50 *μ*m. (b) Cell viability of different group cells analyzed by CCK8 experiment. (c) Cell cycle of different evaluated by flow cytometry. (d) The percentage distribution of cells in each cell cycle phase after different treatment. Data represent the mean ± SD of three experiments, each performed in triplicate. ^*∗∗*^, ^##^, ^∧∧^*p* < 0.01.

TGF-*β*-induced ASMC proliferation is an important reason of airway wall and tissue remodeling [26]. Based on this, primary ASMCs isolated from control and model group were employed to investigate the role of Tetrandrine on cell growth ability of ASMCs. As shown in Figure 2(a), primary ASMCs were authenticated by *α*-actin immunofluorescence detection and the purity was more than 98% in both groups (Figure 2(a)). In CCK8 assay, we found that cells viability of ASMCs from OVA sensitized rats was higher than that in the control group. Once treated with TGF-*β*1, growth ability of ASMCs showed a significant increase compared with the ASMC model group. However, cell viability of AMSCs obviously declined gradually with Tetrandrine treatment in a dose-dependent manner (10, 20, and 40 *μ*g/ml) (Figure 2(b)). Furthermore, the decline in proportion of G0/G1-phase cells and the increase in proportion of S-phase cells in ASMCs model group were further decreased compared with that of the ASMCs model group, while different concentrations of TGF-*β*1 addition obviously induced cell cycle arrest of ASMCs resulting in the enhancement of G0/G1-phase population and the inhibition of S-phase population in a dose-dependent manner compared with the TGF-*β*1 plus ASMCs model group (Figures 2(c)-2(d)). Therefore, these findings prove that Tetrandrine blunts TGF-*β*1-induced ASMCs proliferation in a dose-dependent manner in vitro. Possibly, the inhibition effect of Tetrandrine on ASMCs proliferation contributes to the development of asthma by inhibiting airway remolding.

### 3.3. Tetrandrine Relieves OVA-Evoked Oxidative Stress and the Secretion of Matrix Metalloproteinases

**Figure 3:**
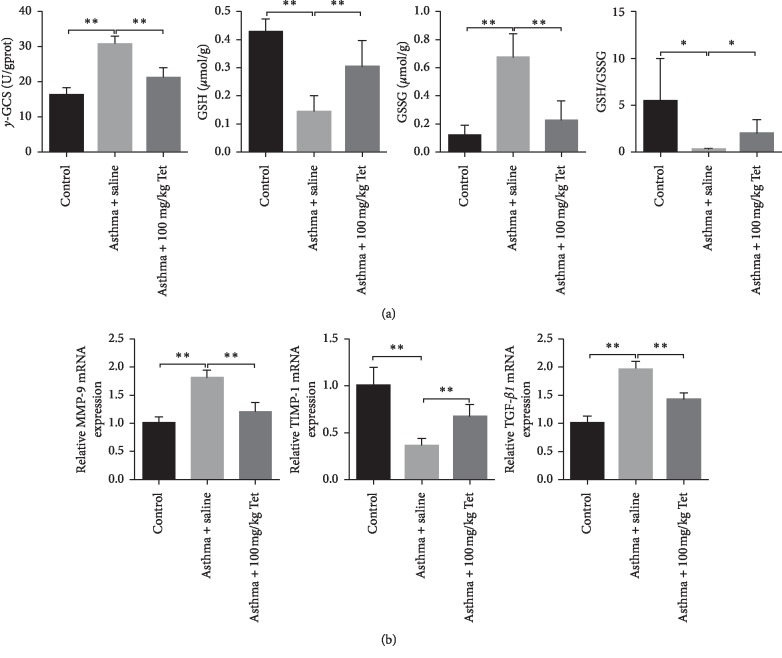
The role of Tetrandrine on oxidative stress. (a) Detection of oxidative stress-related indicators including *γ*-GCS (*p* < 0.01, *F* value = 52.56), GSH (*p* < 0.01, *F* value = 25.12), GSSG (*p* < 0.01, *F* value = 25.79), and the ratio of GSH/GSSG (*p* < 0.01, *F* value = 4.297) by specific kits. (b) Relative mRNA expression of TGF-*β* (*p* < 0.01, *F* value = 62.60), MMP-9 (*p* < 0.05 and 0.01, *F* value = 43.83), and TIMP-1 (*p* < 0.01, *F* value = 31.71) measured by qRT-PCR. Data represent the mean ± SD of three experiments, each performed in triplicate. ^*∗*^*p* < 0.05; ^*∗*^^*∗*^*p* < 0.01.

Increased oxidative stress and ROS have been detected in asthma patients, which act as a key regulator in the process of airway remolding [27, 28]. Since Tetrandrine administration ameliorated airway remolding, it might be involved in the cellular process of oxidative stress in the asthma model. Actually, the expression of *γ*-GCS (a rate-limiting enzyme for glutathione synthesis) in lung tissue of OVA-sensitive rats was eminently enhanced compared with the control group. In contrast, the level of GSH (a powerful antioxidant) was notably declined, but the level of GSSG was obviously promoted in the asthma model group compared to control rats. The ratio of GSH to GSSG which reflected the systemic antioxidant ability was sharply reduced in the asthma group compared with the control group (Figure 3(a)). In comparison with the asthma group, treatment with Tetrandrine strikingly rescued OVA-mediated enhancement of *γ*-GCS and GSSG and restored the inhibition action of OVA on GSH level and the ratio of GSH/GSSG, which suggested that Tetrandrine significantly suppressed OVA-induced oxidative stress (Figure 3(a)). Our data further confirmed that OVA exposure increased the expression of MMP-9 and TGF-*β*1 mRNA in lung tissues of the model group and decreased the level of TIMP-1 mRNA (tissue inhibitors of metalloproteinases). Notably, compared with asthma group, the Tetrandrine-treated group exhibited a prominent reduction of MMP-9 and TGF-*β*1 transcriptional level and a significant promotion of TIMP-1 mRNA (Figure 3(b)). Collectively, these results indicate that Tetrandrine reverses airway remodeling potentially by suppressing oxidative stress and subsequently the secretion of MMPs and TGF-*β*1 expression.

### 3.4. Tetrandrine Blunts Oxidative Stress via Affecting Nrf-2/HO-1 Signaling In Vivo

**Figure 4:**
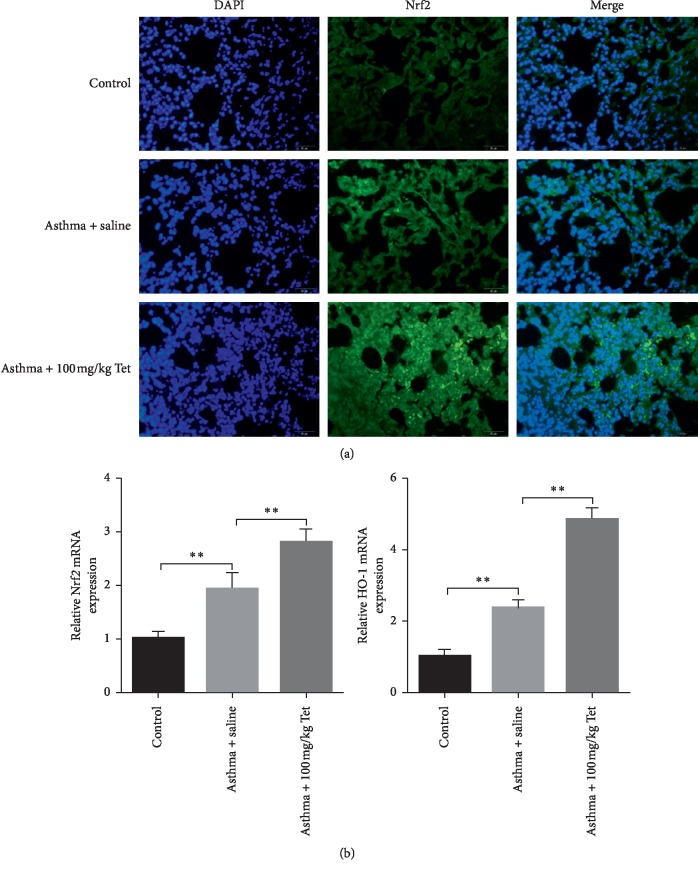
Impacts of Tetrandrine on Nrf-2/HO-1 signaling in vivo. (a) The expression of Nrf-2 protein determined with IF assay. (b) Relative mRNA expression of Nrf-2 (*p* < 0.01, *F* value = 50.42) and HO-1 (*p* < 0.01, *F* value = 191.1) analyzed by qRT-PCR. Scale bar: 50 *μ*m. ^*∗*^*p* < 0.05; ^*∗*^^*∗*^*p* < 0.01.

Nuclear erythroid factor 2-related factor 2 (Nrf-2) is involved in the process of oxidative stress-induced lung damage [29, 30]. In control rats, we observed that Nrf-2 presented low level and localized in the cytoplasm. Under the stimulation of OVA, Nrf-2 was freed and moderately accumulated in nucleus in rat lung tissue. However, there showed robust nucleus activation of Nrf-2 in Tetrandrine-treated group compared to the model group (Figure 4(a)). Besides, relative mRNA expression of Nrf-2 also showed a moderate increase in model rats which presented a higher level in Tetrandrine administration group (Figure 4(b)). Additionally, OVA treatment induced the increase of HO-1 expression whose effect was enlarged by the administration of Tetrandrine (Figure 4(b)). Based on the inhibition role of Nrf-2 activation on oxidative stress, we thought that nucleus activation of Nrf-2 and the increase of Nrf-2 and HO-1 in model rats might be protective adaptation mechanism of rats. And Tetrandrine treatment further activated Nrf-2 and HO-1 transcription in lung tissues, leading to the boom of oxidative stress decline. These data suggest that Tetrandrine possibly relieves oxidative stress by activating Nrf-2/HO-1 signaling.

### 3.5. Tetrandrine Reverses TGF-*β*1-Mediated Enhancement of CysLT1/CysLTR1 and the Activation of Nrf-2/HO-1 Pathway In Vitro

**Figure 5:**
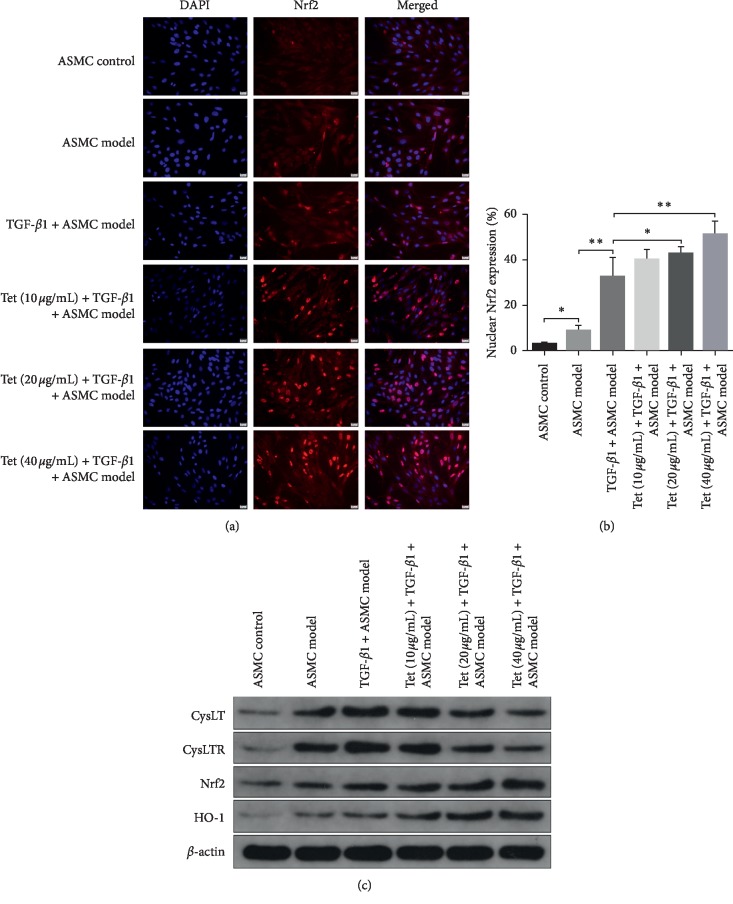
Effects of Tetrandrine on Nrf-2 nuclear translocation and expression of CysLT1 and CysLTR1in vitro. (a) Nrf-2 protein expression of ASMCs determined by immunofluorescence assay. (b) Quantitative analysis of nuclear Nrf-2 protein expression after different treatments. (c) CysLT1, CysLTR1, Nrf-2, and HO-1 protein levels were determined with western blot. *β*-actin was used as a loading control. Multiple images were taken, and the representative one is presented. Scale bar: 20 *μ*m. ^*∗*^*p* < 0.05, ^*∗∗*^*p* < 0.01.

To elucidate the role of Tetrandrine on oxidative stress-mediated airway remodeling in vitro, primary ASMCs were employed to measure the changes in the Nrf-2/HO-1 pathway. As indicated in Figure 5(a), primary AMSCs from control rats presented low expression of Nrf-2, most of which existed in the cytoplasm. In AMSCs isolated from the model group, nucleus activation of Nrf-2 showed a moderate enhancement compared to that of control cells (Figure 5(a)). When treated with TGF-*β*1, nuclear translocation of Nrf-2 was further promoted in a dose-dependent manner of TGF-*β*1 concentration compared with AMSCs extracted from model rats (Figure 5(a)). Quantitative analysis showed that nuclear cells of Nrf-2-positive AMSCs in cells from model rats were upregulated compared to control cells. TGF-*β*1 exposure robustly promoted the number of Nrf-2-positive cells in comparison with the ADSC model group (Figure 5(b)). However, treatment with Tetrandrine notably increased the rate of Nrf-2-positive cells in nucleus at the concentration of 20 and 40 *μ*g/ml, though 10 *μ*g/ml of Tetrandrine did not show significant difference compared with the TGF-*β*1 plus AMSCs model group (Figure 5(b)). Additionally, inflammation and oxidative stress pathway-associated proteins were also evaluated in in vitro experiments. As observed in Figure 5(c), the expressions of CysLT1 and CysLTR1 were gradually promoted in primary ASMCs of OVA sensitive rats which showed more obvious enhancement when TGF-*β*1 was added. However, Tetrandrine inhibited the expression of CysLT1 and CysLTR1 showing stronger inhibitory effect under the condition of high concentration of Tetrandrine (20 and 40 *μ*g/ml) (Figure 5(c), the top two bands). Simultaneously, the proteins levels of Nrf-2 and HO-1 were accelerated based on the adaptation mechanism in the AMSC model group. TGF-*β*1 addition also significantly enhanced Nrf-2 and HO-1 protein levels compared with the ASMCs model group. Interestingly, treatment with Tetrandrine (10, 20, and 40 *μ*g/ml) notably promoted the expressions of Nrf-2 and HO-1 in a dose-dependent manner (Figure 5(c)). These results demonstrate that Tetrandrine potentially remits inflammation and oxidative stress by inhibiting CysLT1 and CysLTR1 expressions and activating the Nrf-2/HO-1 signaling pathway.

## 4. Discussion

Airway remodeling is one of the main pathologies of asthma, and effective therapy on asthma has not been established [31]. Current clinical drugs including long-acting *β*2-agonist, bronchodilators, and inhaled corticosteroid have little effect on airway remodeling [32]. Therefore, investigating effective drugs targeting airway remodeling has a great significance in clinical treatment of asthma. The present study revealed that Tetrandrine attenuated the airway remodeling of OVA-sensitive rat and ASMCs via affecting oxidative stress which possibly provided powerful evidence for the treatment of asthma.

Airway remodeling refers to architecture changes of the airway including subepithelial fibrosis, hyperplasia of ASMCs, and excessive extracellular matrix (ECM), which are the main reasons of physiological lung function decline [33]. Airway remodeling contains a variety of pathological factor, including allergen, growths factor, ROS, and proinflammatory factor [34]. For example, cysteinyl leukotrienes (CysLTs), including LTC4, LTD4, and LTE4, exert their proinflammatory effects through binding to CysLT1 and its receptors in asthmatic [35]. Cysteinyl leukotrienes and TGF-*β* promote airway remodeling by enhancing proliferation and migration of ASMCs, which obviously accelerates airway wall thickening [36, 37]. Regulation of ASM hyperplasia or TGF-*β* activity has been considered potential therapeutic targets abrogating airway remodeling [38]. In the present study, we found that Tetrandrine decreased alveolar inflammatory infiltration and airway remolding along with the inhibition of CysLT1 and CysLTR1 in OVA sensitive rats. In primary ASMCs, Tetrandrine also disturbed TGF-*β*1-induced cell proliferation of AMSCs in vitro. However, others CysLTs and airway epithelium also play pivotal role in inflammatory response of asthma [39]. Whether Tetrandrine mediated the improvement of airway remolding and inflammation response is involved with other CysLTs which needs further research. Additionally, epithelial-mesenchymal transition (EMT), as one of the most important mechanisms of airway inflammation, is considered a promoter of airway remodeling during asthma [40]. Herein, we discovered that the increase of mesenchymal markers *α*-smooth muscle actin (*α*-SMA) in asthma rat was inhibited by administration of Tetrandrine, which suggested that Tetrandrine mediated the improvement of airway remodeling partly through regulating the EMT process.

Oxidative stress-induced airway remodeling has attracted much attention of researchers in recent years. Oxidative stress activates immunoreactivity of NF-*κ*B by promoting the expression of inflammatory factors of the lung [41, 42]. Oxidative stress mediated the activation of TGF-*β*1 results in hyperreactivity of AMSCs and epithelium injury [43, 44]. Extracellular matrix (ECM) is also a hallmark in the process of oxidative stress-evoked airway remodeling. Oxidative stress activation mediated the production of matrix metalloproteinases (MMPs) which also facilitates to pulmonary fibrosis [45], ECM, and tissue architecture, which are able to result in the process of airway remodeling [46]. It has been demonstrated that oxidative stress can activate TGF-*β* and MMPs genes transcription, which are involved in the development of pulmonary fibrosis [47, 48]. Our results indicated that Tetrandrine reduced the expression of oxidative stress-related factors, TGF-*β*1, and MMPs in the lung of asthmatic rats. This indicates that Tetrandrine improves antioxidant defense and reduces the expression of downstream MMPs. TGF-*β*1 has been confirmed as a “master switch” in the procedure of EMT [5]. Given that Tetrandrine treatment decreased the level of TGF-*β*1 in asthma rats, it was further confirmed that Tetrandrine mediated the inhibition of the EMT process in asthma.

Once stimulated by oxidative stress, Nrf-2 enters into the nucleus and activates the expression of HO‐1 (an antioxidative modulator) leading to the remission of oxidative stress [49]. The Nrf-2/HO-1 pathway is a predominant defensive mechanism to counter the oxidative damage [50]. Activated Nrf-2 resides in nucleus leading to the activation of HO-1, peroxiredoxin (Prxs), and glutathione peroxidase 1 (GPx1) [51]. The expression of MMP-9 was attenuated by diallyl-disulfide-mediated Nrf-2/HO-1 pathway activation in allergic asthma [52]. The results of the present study demonstrated that Tetrandrine promoted the nuclear activation of Nrf-2 and subsequent increase of HO-1, indicating that Tetrandrine activated the antioxidant defensive mechanism by upregulating Nrf-2/HO-1 signal transduction. Interestingly, primary cells of AMSC model and cells of TGF-*β*1 plus ASMCs model also presented a moderate increase of Nrf-2 expression in nucleus compared with normal control, which is possibly the compensatory effect of the lung under the condition of immense oxidative pressure. Both Nrf-2 and TGF-*β*1 are upregulated in the process of airway remodeling in asthma [53]. There is a crosstalk between Nrf-2/HO-1 and TGF-*β*1/Smad pathways in prostatic deficits such as prostatic oxidative stress, inflammation, and prostatic EMT [54]. Possibly, Tetrandrine mediated the improvement of asthma through regulating oxidative stress- and inflammation-evoked airway remodeling by affecting TGF-*β*1/Nrf-2/HO-1 signaling cascades.

## 5. Conclusion

In summary, this study adequately demonstrated that Tetrandrine reversed the inflammatory response and airway remodeling in vivo and decreased TGF-*β*1-induced promotion of cell viability of AMSCs in vitro by stimulating Nrf-2/HO-1 signaling. Therefore, Tetrandrine may be a novel and effective drug candidate for asthma treatment in clinic.

## Figures and Tables

**Table 1:** Primer sequences for qRT-PCR.

Gene	Froward (5′-3′)	Reverse (5′-3′)
HO-1	CAGAGTCCCTCACAGACAGAGT	TGAACTAGTGCTGATCTGGGATTTT
MMP-9	GCAAACCCTGCGTATTTCCATT	GCGATAACCATCCGAGCGAC
TIMP-1	TAAAGCCTGTAGCTGTGCCC	CATAACGCTGGTATAAGGTGGTC
Nrf2	TCAGCGACGGAAAGAGTATGA	CCACTGGTTTCTGACTGGATGT
TGF-*β*1	GAGAGCCCTGGATACCAACTACTG	GTGTGTCCAGGCTCCAAATGTAG
GAPDH	GGTGGACCTCATGGCCTACAT	GCCTCTCTCTTGCTCTCAGTATCCT

## Data Availability

The data used to support the findings of this study are included within the article.
