# Erratum to “Tetrandrine Ameliorates Airway Remodeling of Chronic Asthma by Interfering TGF-*β*1/Nrf-2/HO-1 Signaling Pathway-Mediated Oxidative Stress”

**DOI:** 10.1155/2020/6958283

**Published:** 2020-02-21

**Authors:** Yiping Lin, Jingchan Yao, Meiling Wu, Xiaoqian Ying, Mingxing Ding, Yanli Wei, Xiaoyan Fu, Wei Feng, Yunguang Wang

**Affiliations:** ^1^Department of Pediatrics, School of Medicine, Jinhua Polytechnic, Jinhua 321007, Zhejiang, China; ^2^Department of Pharmacology, School of Medicine, Jinhua Polytechnic, Jinhua 321007, Zhejiang, China; ^3^Department of Histologic, School of Medicine, Jinhua Polytechnic, Jinhua 321007, Zhejiang, China; ^4^Department of Radiation Oncology, Institute of Cancer Research and Basic Medical Sciences of Chinese Academy of Sciences, Cancer Hospital, University of Chinese Academy of Sciences, Zhejiang Cancer Hospital, Hangzhou 310022, Zhejiang, China; ^5^Institute of Nuclear-Agricultural Sciences, Zhejiang University, Hangzhou 310058, Zhejiang, China

In the article titled “Tetrandrine Ameliorates Airway Remodeling of Chronic Asthma by Interfering TGF-*β*1/Nrf-2/HO-1 Signaling Pathway-Mediated Oxidative Stress” [1], the authors Yunguang Wang and Wei Feng were not listed as the corresponding authors. The updated corresponding authors are listed above.
